# Adaptable covalently cross-linked fibers

**DOI:** 10.1038/s41467-023-37850-w

**Published:** 2023-04-18

**Authors:** Hui Tan, Luzhi Zhang, Xiaopeng Ma, Lijie Sun, Dingle Yu, Zhengwei You

**Affiliations:** 1 Respiratory Department, Shenzhen Children’s Hospital, 518038 Shenzhen, China; 2 State Key Laboratory for Modification of Chemical Fibers and Polymer Materials, College of Materials Science and Engineering, Institute of Functional Materials, Research Base of Textile Materials for Flexible Electronics and Biomedical Applications (China Textile Engineering Society), Shanghai Engineering Research Center of Nano-Biomaterials and Regenerative Medicine, Donghua University, 201620 Shanghai, China

**Keywords:** Polymers, Mechanical properties, Polymers

## Abstract

Fibers, with over 100 million tons produced each year, have been widely used in various areas. Recent efforts have focused on improving mechanical properties and chemical resistance of fibers via covalent cross-linking. However, the covalently cross-linked polymers are usually insoluble and infusible, and thus fiber fabrication is difficult. Those reported require complex multiple-step preparation processes. Herein, we present a facile and effective strategy to prepare adaptable covalently cross-linked fibers by direct melt spinning of covalent adaptable networks (CANs). At processing temperature, dynamic covalent bonds are reversibly dissociated/associated and the CANs are temporarily disconnected to enable melt spinning; at the service temperature, the dynamic covalent bonds are frozen, and the CANs exhibit favorable structural stability. We demonstrate the efficiency of this strategy via dynamic oxime-urethane based CANs, and successfully prepare adaptable covalently cross-linked fibers with robust mechanical properties (maximum elongation of 2639%, tensile strength of 87.68 MPa, almost complete recovery from an elongation of 800%) and solvent resistance. Application of this technology is demonstrated by an organic solvent resistant and stretchable conductive fiber.

## Abstract (web summary)

Covalent cross-linking can be used to improve the mechanical properties of fibers, but can be complicated to prepare and process. Here, the authors report fibers prepared with dynamic covalent bonds, which can reversibly dissociate to enable processing, while maintaining improved mechanical properties in the final material.

## Introduction

Fibers are ubiquitous and play a vital role in various fields including our daily life (e.g., clothing, construction industries, automotives) and emerging applications (e.g., medical devices, energy storage, wearable electronics, space exploration)^1–4^. Existing fibers are mostly made of thermoplastics such as polyolefins, polyesters, and polyamides. Thermoset fibers are attractive because of their robust mechanical properties (e.g., tensile strength, elastic recovery) and chemical/thermal resistance resulting from their covalently cross-linked structures^5^. However, traditional covalently cross-linked polymers such as epoxy resin and vulcanized rubber cannot be processed in the same way as thermoplastic polymers due to their permanent three-dimensional covalent networks, which is insoluble and infusible^6^. The preparation of covalently cross-linked fibers remains a significant challenge. There are few reports on covalently cross-linked fibers. Yang’s group prepared covalently cross-linked liquid crystal elastomer fibers by a two-step method of mold processing and UV light post-curing^7^. Zhang’s group reported a covalently cross-linked azobenzene-based polymer fiber by immersing the melt-spun fibers in a solution containing a cross-linking agent followed by washing and drying^8^. These methods are complicated and cannot achieve continuous preparation of covalently cross-linked fibers. Recently, covalently cross-linked nonwoven fabrics has been reported^9–11^. However, their applications are limited compared to traditional fibers. The continuous preparation of covalently cross-linked gel fibers through the combination of wet spinning technology and in situ UV light curing have been recently realized^5,12,13^. Nevertheless, gel fibers have not been widely used for real applications. Thus, it is urgent to develop a convenient and efficient strategy for the preparation of covalently cross-linked fibers.

Covalent adaptable networks (CANs) are a class of polymers cross-linked by reversible covalent bonds such as disulfide bonds, oxime-urethane bonds, Diels-Alder adducts, and so on^14–18^. CANs can change the topology of the networks through the reversible exchange of dynamic covalent bonds under external stimuli (e.g., heat, light). Therefore, CANs possess plasticity and reprocessability similar to traditional thermoplastics.

**Fig. 1:**
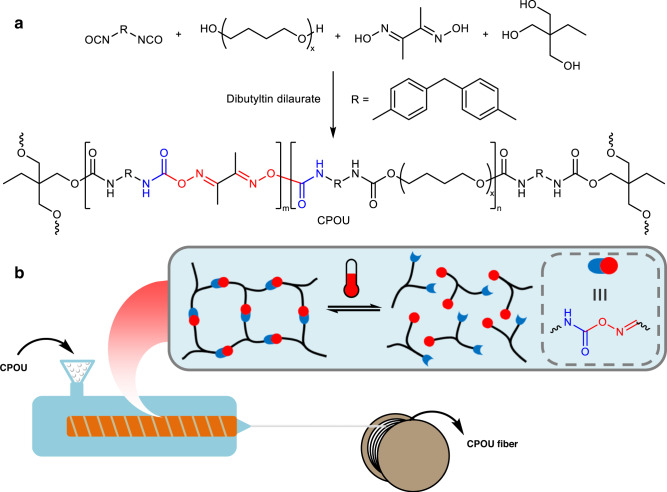
Design and fabrication of CPOU fiber. **a** Molecular design of covalently cross-linked CPOU. **b** Schematic of the melt-spinning process and corresponding molecular evolution of CPOU.

In this work, we propose a strategy to prepare adaptable covalently cross-linked fibers by melt spinning of CANs. Compared with dry spinning and wet spinning, melt spinning is simple and cost-efficient. The process of melt spinning does not require any coagulation baths or solvents, and does not produce any emissions and residues. At processing temperature, the reversible dissociation/association of dynamic covalent bonds is accelerated, resulting in a rapid decrease of polymer viscosity to enable melt spinning. At the service temperature, the dynamic covalent bonds are frozen, and CANs exhibit robust mechanical properties and solvent resistance consistent with traditional thermosets. We demonstrate this strategy via covalently cross-linked poly(oxime-urethane) (CPOU) fibers with high stretchability, excellent elastic recovery, and solvent resistance (Fig. 1b).

## Results

### Material design and characterizations

The oxime-urethane based covalently cross-linked CPOU was readily synthesized by one-pot polyaddition of commercially available polytetramethylene ether glycol, diphenylmethane diisocyanate, dimethylglyoxime and trimethylolpropane, in the presence of catalyst dibutyltin dilaurate (Fig. 1a). Polytetramethylene ether glycol was selected as soft segment because of its flexible polymer chain. The reaction of diphenylmethane diisocyanate and chain extender dimethylglyoxime produced reversible oxime-urethane bonds^19–24^. The integration of triple-functional trimethylolpropane yielded covalently cross-linked networks.

**Fig. 2:**
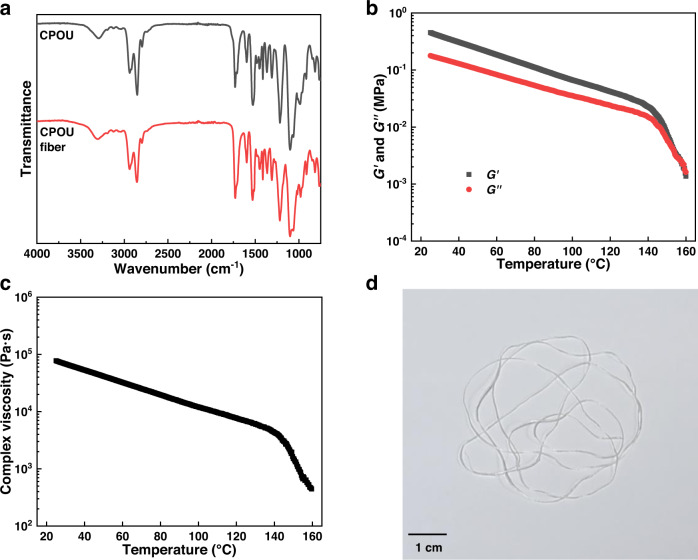
Spinnability of CPOU. **a** FTIR spectra of as synthesized CPOU and CPOU fiber. **b** Temperature-dependent curves of storage modulus and loss modulus of CPOU. **c** Temperature-dependent curve of complex viscosity of CPOU. **d** Photograph of CPOU fiber.

The structure of CPOU was characterized via Fourier transform infrared (FTIR) spectroscopy in the attenuated total reflectance mode. The FTIR spectra (Fig. 2a) present peaks at 3297 and 1728 cm^−1^ corresponding to the N–H and C = O bonds, respectively, indicating the successful formation of urethane groups. The presents peaks at 984 cm^−1^ corresponding to the N–O bonds. The indistinct peak corresponding to the isocyanate group at approximately 2270 cm^−1^ indicated the full conversion of the diphenylmethane diisocyanate monomers. These results confirmed the successful synthesis of CPOU.

The thermal properties and thermomechanical behavior of CPOU were investigated by thermogravimetric analysis (TGA), differential scanning calorimetry (DSC), and dynamic mechanical analysis (DMA). TGA result showed a good thermal stability of CPOU with a weight loss of 5% at 278 °C (Supplementary Fig. 1). No crystallization nor melting peaks were detected in the DSC curve of the CPOU from −50 to 150 °C (Supplementary Fig. 2). DMA revealed its glass-transition temperature (*T*_g_) of −33.8 °C (Supplementary Fig. 3).

Next, the suitability of CPOU for melt spinning was studied in detail by rheological analysis. The storage modulus (*G*′), loss modulus (*G*″), and complex viscosity of CPOU continuously decreased with increasing temperature (Fig. 2b). The temperature of intersection between *G*′ and *G*″ for CPOU was 153 °C, indicating the transition of networks from solid to liquid state and implying the feasibility of stable melt spinning at moderate temperature. At 153 °C, the complex viscosity of CPOU decreased to 1000 Pa·s, which was suitable for melt spinning^25^. TPOU with the same backbone structure as CPOU but without the covalently cross-linked structure was synthesized as a control sample. The solid-liquid transition temperature of CPOU (153 °C) was higher than that of TPOU (128 °C; Supplementary Fig. 4). Interestingly, the complex viscosities of CPOU (153 °C) and TPOU (149 °C) went down to 1000 Pa s at similar temperatures (Supplementary Fig. 5). The main reason for these results was the rapid reversible dissociation/association of a large number of oxime-urethane bonds, and resulting in the temporarily disconnected networks. Thus, the complex viscosities of both CPOU (1.5 × 10^3 ^Pa s) and TPOU (9.6 × 10^2^ Pa s) at 150 °C were significantly lower than that of the traditional thermoplastic polyurethanes without dynamic covalent bonds (1.6 × 10^4^−1.0 × 10^5^ Pa s)^25,26^. The melt spinning temperature of traditional thermoplastic polyurethanes was usually higher than its decomposition temperature, resulting in the degradation of the polymer during the spinning process. Overall, our proposed strategy of direct melt spinning by CANs is not only expected to be suitable for the preparation of covalently cross-linked fibers, but also provides an idea for the melt spinning of polymers with high melting temperature such as polyurethane.

The Arrhenius equation was applied to deduce the apparent activation energies (*E*_a_) from the results of the temperature-dependent stress relaxation tests (Supplementary Figs. 6–9). The *E*_a_ of CPOU (119.7 kJ mol^−1^) was significantly higher than that of TPOU (29.5 kJ mol^−1^), indicating the higher structural stability of CPOU than that of TPOU.

In view of the above factors, CPOU fiber was melt spun at 155 °C (Fig. 2d). The FITR spectrum (Fig. 2a), TGA curve (Supplementary Fig. 1), and DSC curve (Supplementary Fig. 2) of CPOU fiber were essentially identical to those of CPOU, indicating that the structure of CPOU remained unchanged after melt spinning.

### Mechanical properties

**Fig. 3:**
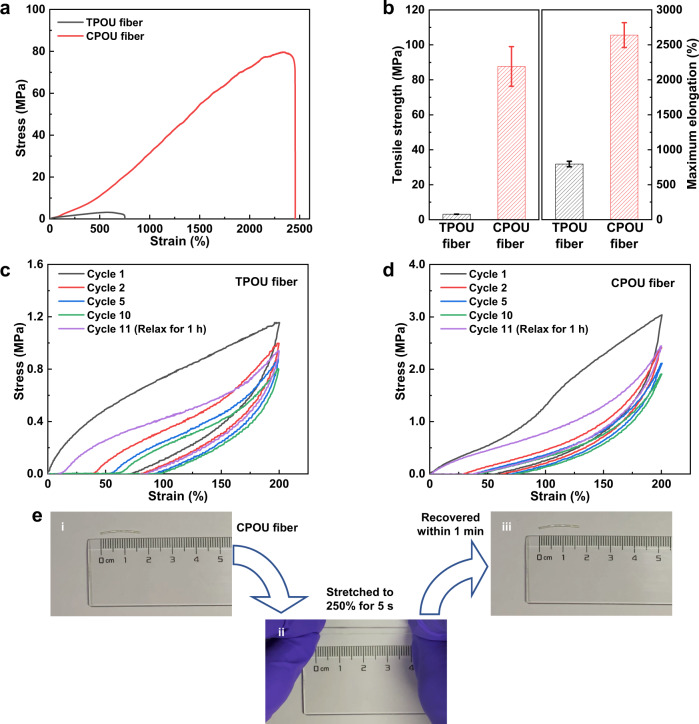
Mechanical properties of TPOU and CPOU fibers. **a** Typical tensile stress-strain curves. **b** Comparison of their tensile strength and maximum elongation (error bars: standard deviations). **c**, **d** Cyclic tensile curves of **c** TPOU fiber and **d** CPOU fiber at 200% strain. There was no break between each consecutive cycle for the first 10 cycles. After being allowed the samples to relax for 1 h at room temperature, the 11th cyclic tensile test was performed. **e** Photographs of (i) original, (ii) 2.5 times stretched, and (iii) 1 min recovered (at room temperature) CPOU fiber.

Next, the mechanical properties of CPOU and TPOU fibers were studied in details (Fig. 3a, b). The tensile strength of CPOU fiber (87.68 ± 11.34 MPa) was more than 28 times that of TPOU fiber (3.13 ± 0.05 MPa), while the maximum elongation of CPOU fiber (2639% ± 178%) was 3.3 times that of TPOU fiber (795% ± 40%). The toughness of CPOU fiber (1187 ± 227 MJ m^−3^) was more than 74 times that of TPOU fiber (16 ± 1 MJ m^−3^). These results indicated that the covalently cross-linked structure could significantly improve the mechanical properties of fibers.

Owing to the dynamic nature of the CPOU networks, CPOU fibers can be recycled and melt-spun again. Compared with original CPOU fibers, the mechanical properties of the recycled CPOU fibers were basically maintained (Supplementary Fig. 10), demonstrating the recyclability of CPOU fibers.

The polymeric networks, preparation methods, and mechanical properties of reported fibers were summarized in Supplementary Table 1. For covalently cross-linked fibers: some gel fibers could be continuously prepared by microfluidic spinning/wet-spinning and post-crosslinking, but their mechanical properties (tensile strength ~1 MPa) might be difficult to meet the needs of many practical use scenarios; liquid crystal polymer fibers were prepared by wet-spinning/mold-processing and post-crosslinking, so it was difficult to achieve continuous preparation. For non-covalently cross-linked fibers (especially polyurethane fibers), they usually showed appreciable tensile strength (34–87 MPa) but limited maximum elongation (320–520%). Most of polyurethane fibers were prepared by wet-spinning, which required the use of organic solvents (e.g., dimethylformamide) and additional post-treatment processes (e.g., solvent removal). In this work, the spinning process of adaptable covalently cross-linked CPOU fibers did not require solvents and achieved continuous preparation. In addition, the tensile strength (87.7 MPa) of CPOU fibers exceeded most of the reported fibers, and showed high maximum elongation (2639%).

To demonstrate the elastic performance of CPOU fiber, repeated cyclic tensile tests at a constant strain of 200% were performed (Fig. 3c, d). There was no break between each consecutive cycle for the first ten cycles. After being allowed the samples to relax for 1 h at room temperature, the 11th cyclic tensile test was performed. TPOU fiber was used as a control sample. The residual strains of CPOU fiber at the beginning of the 2nd and 10th cycle were 29% and 44%, respectively, which were significantly smaller than that of TPOU fiber (40% and 96%). At the beginning of the 11th cycle, the residual strain of the CPOU fiber was negligible, while the TPOU fiber still had a significant residual strain of 10%. These results confirmed that CPOU fiber had much higher elastic recoverability than TPOU fiber because of its cross-linked structure. As a demonstration, CPOU fiber was stretched to 2.5 times its original length for 5 s and almost recovered to its original dimension after relaxing for 1 min at room temperature (Fig. 3e). Furthermore, the CPOU fiber was stretched to 8 times its original length for 5 s and almost recovered to its original dimension after relaxing for 1 h at room temperature (Supplementary Fig. 11), demonstrating its excellent stretchability and resilience.

### Solvent resistance

**Fig. 4:**
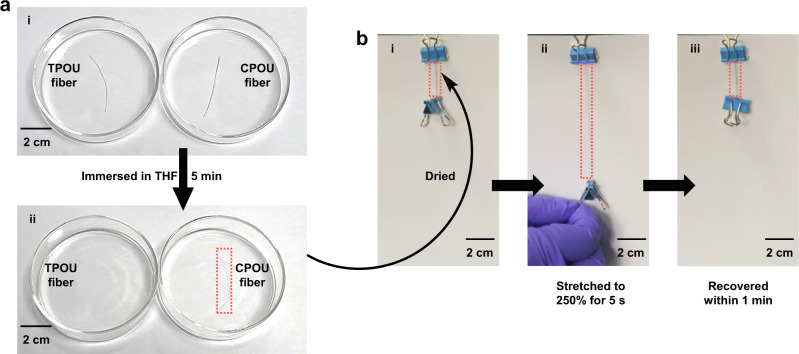
Solvent resistance of TPOU and CPOU fibers. **a** Photographs of dissolution tests of TPOU and CPOU fibers. (i) Fibers were immersed in THF at room temperature; (ii) after 5 min, TPOU fiber dissolved, while CPOU fiber kept intact. **b** Photographs of dried CPOU fiber after swelling with THF: (i) lifting an object, (ii) 2.5 times stretched, and (iii) recovered within 1 min.

The solvent resistance of materials is an important characteristic for practical use. However, most existing fibers have limited organic solvent resistance due to their non-covalently cross-linked structures^27^. As designed, CPOU fiber is expected to have excellent organic solvent resistance. According to dissolution experiments, CPOU fibers were insoluble in common solvents, and the swelling ratios were summarized in Supplementary Table 2. For demonstration, CPOU and TPOU fibers were immersed in tetrahydrofuran (THF) at room temperature. After 5 min, CPOU fiber only swelled and maintained its original fiber shape, while the TPOU fiber completely dissolved (Fig. 4a). Moreover, after the CPOU fibers were immersed in organic solvents with different polarities for 5 min and dried, the mechanical properties of the CPOU fibers were basically maintained (Supplementary Fig. 12), demonstrating the solvent resistance of the CPOU fibers. Furthermore, the dried CPOU fiber after swelling with THF could almost recover within 1 min after being stretched to 2.5 times its original length for 5 s (Fig. 4b). Besides, the dried CPOU fiber after swelling with THF was recovered (residual deformation: 20%) within 1 min after being stretched to 4.5 times its original length for 5 s (Supplementary Fig. 13). These results indicated the excellent organic solvent resistance of the CPOU fibers.

### Organic solvent resistant and stretchable conductive fiber

**Fig. 5:**
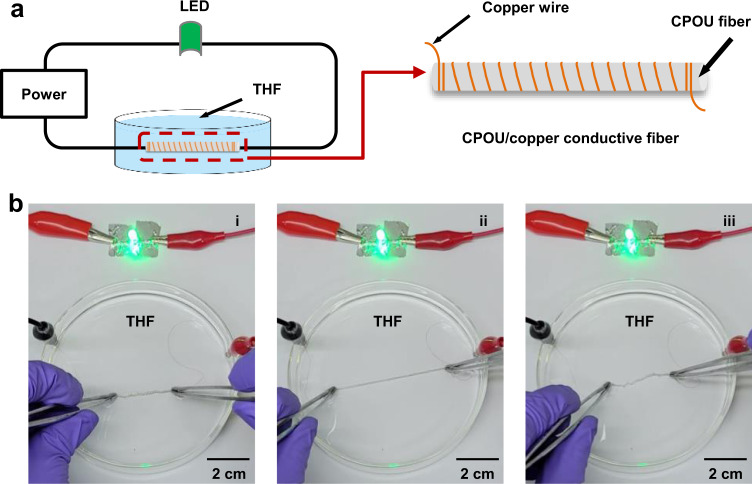
Organic solvent resistant and stretchable conductive fiber made of CPOU. **a** Schematic of the electronic circuit consisting of the CPOU/copper conductive fiber. **b** Demonstration of the organic solvent resistant and stretchable properties for the CPOU/copper conductive fiber in series with an LED. (i) CPOU/copper conductive fiber immersed in THF, (ii) 2.5 times stretched for 5 s, and (iii) recovered within 1 min.

Stretchable electronic materials are highly desirable for booming flexible electronics^28–31^. With the continuous expansion of application fields, stretchable electronic materials need to face various complex service environments including organic solvents. However, the fabrication of organic solvent resistant and stretchable conductor remains a great challenge. As a demonstration, we fabricated a CPOU/copper conductive fiber, which could efficiently light up a light-emitting diode (LED) lamp in a circuit applied voltage of 3 V (Fig. 5a). After being immersed in THF for 5 min, CPOU/copper fiber maintained high conductivity, stretchability and elasticity with an immediate (within 1 min) recovery to 1.06 times its original length after being 2.5 times stretched for 5 s (Fig. 5b).

## Discussion

In summary, we established a simple and efficient strategy to continuously prepare adaptable covalently cross-linked fibers based on dynamic oxime-urethane bonds. The key is that the reversible dissociation/association of dynamic covalent bonds is accelerated at processing temperature, resulting in a rapid drop in polymer viscosity to enable melt spinning; dynamic covalent bonds are frozen at the service temperature, forming a stable network contributing to structural stability and high performance. More importantly, the large number of dynamic oxime-urethane bonds endow CPOU with suitable viscosity for melt spinning below the degradation temperature, which is difficult to achieve even in traditional thermoplastic polyurethanes. CPOU fiber exhibited robust mechanical properties and organic solvent resistance. The potential applications of CPOU fiber have been demonstrated as an organic solvent resistant and stretchable conductive fiber, which will broaden the application scenarios of booming stretchable electronic devices. The proposed strategy would also be applicable to widely available CANs based on other dynamic covalent systems (e.g., disulfide bonds, imine bonds, Diels-Alder adducts), to develop diverse adaptable covalently cross-linked fibers with unique properties. This work will not only promote the development of covalently cross-linked fibers, but also provide an idea for efficient melt spinning of high-performance polymers with melting temperature higher than decomposition temperature, and enable a series of applications.

## Methods

### Materials

All reagents were used as received without further purification unless otherwise noted. Polytetramethylene ether glycol (*M*_n_ ~1000 g mol^−1^) and dibutyltin dilaurate (95%) were purchased from Aladdin Chemical Co., Ltd. (Shanghai, China). Diphenylmethane diisocyanate (a 50∶50 mixture of 2,4′- and 4,4′-diphenylmethane diisocyanate, 99.6%) was purchased from Wanhua Chemical (Yantai, China). Dimethylglyoxime (98%) was purchased from Sinopharm Chemical Reagent (Shanghai, China). Trimethylolpropane (98%) and anhydrous tetrahydrofuran were purchased from J&K Chemical Technology (Beijing, China). The purchased polytetramethylene ether glycol was dehydrated under vacuum at 110 °C for 2 h and then cooled to 25 °C before use.

### Synthesis of TPOU

Polytetramethylene ether glycol (8.000 g, 8.00 mmol), dimethylglyoxime (0.348 g, 3.00 mmol), and diphenylmethane diisocyanate (2.766 g, 11.05 mmol) were dissolved in 15 mL of tetrahydrofuran in a round-bottomed flask. Dibutyltin dilaurate (0.050 g) was added to the mixture and reacted for 20 min under magnetic stirring at 60 °C in an N_2_ atmosphere. Thereafter, the reaction mixture was poured into a polytetrafluoroethylene mold at 60 °C, and the temperature was gradually increased to 85 °C over 12 h. Finally, TPOU was obtained under vacuum for 48 h at room temperature.

### Synthesis of CPOU

Polytetramethylene ether glycol (8.000 g, 8.00 mmol), dimethylglyoxime (0.348 g, 3.00 mmol), diphenylmethane diisocyanate (3.066 g, 12.25 mmol), and trimethylolpropane (0.107 g, 0.8 mmol) were dissolved in 15 mL of tetrahydrofuran in a round-bottomed flask. Dibutyltin dilaurate (0.050 g) was added to the mixture and reacted for 20 min under magnetic stirring at 60 °C in an N_2_ atmosphere. Thereafter, the reaction mixture was poured into a polytetrafluoroethylene mold at 60 °C, and the temperature was gradually increased to 85 °C over 12 h. Finally, CPOU was obtained under vacuum for 48 h at room temperature.

### Preparation of fibers

The preparation of CPOU fiber was conducted by single screw extruder (Wellzoom E) at 155 °C with screw speed of 50 rpm. The extrusion speed was 3 m min^−1^, draw ratio was set as 2. The extrusion temperature of TPOU was 150 °C, and other parameters were the same as CPOU.

### General characterization

All the tests were performed at room temperature (25 °C), unless specified otherwise. The Fourier-transform infrared (FTIR) spectra were recorded using the Nicolet 8700 spectrometer equipped with an attenuated total reflectance accessory from Thermo Fisher Scientific. Thermogravimetric analysis (TGA) was performed on a TG 209 F1 thermogravimetric analyzer (NETZSCH) from 30 to 600 °C at a heating rate of 20 °C min^−1^ under nitrogen atmosphere. Differential scanning calorimetry (DSC) were performed on a Q20 differential scanning calorimeter from TA Instruments. Samples were heated from 25 to 160 °C, cooled to −70 °C, and reheated to 160 °C at a rate of 10 °C min^−1^ under a nitrogen atmosphere. All the data were obtained from the second heating curves. Dynamic mechanical analyses (DMA) were performed at a DMA1 (METTLER TOLEDO) dynamic mechanical analyzer. Rectangular samples were tested at a frequency of 1 Hz and a strain of 0.1%. Heating ramps of 5 °C min^−1^ were applied from −100 to 100 °C. The glass transition temperatures (*T*_*g*_) were estimated from the maximum loss factor. Rheological experiments and temperature-dependent stress-relaxation experiments were performed using the ARES-G2 rotational rheometer with 8 mm diameter parallel plates from TA Instruments. Temperature sweeps were performed from 25 to 160 °C at 0.5% stain and 1 Hz. The temperature-dependent stress-relaxation experiments were performed in the strain-control mode (1% strain). The relaxation modulus (*G*) was normalized to the initial modulus (*G*_0_). The characteristic relaxation time (τ*) was defined as the time required for *G*/*G*_0_ to reach 1/*e*, according to the following exponential decay function: *G*(*t*) = *G*_0_ exp (-*t*/τ*). The apparent activation energy (*E*_a_) was calculated using the Arrhenius equation, ln τ*(*T*) = ln *τ*_0_ + *E*_a_ /*RT*. Here, *τ*_0_, *R*, and *T* represent the characteristic relaxation time at an infinite temperature, universal gas constant, and absolute temperature, respectively. The swelling ratios were determined by soaking the sample (original weight, *W*_*0*_) in specified solvent. The weight of the swollen sample was signed as *W*_*1*_. The swelling ratios were calculated from the following equations: swelling ratio = *W*_1_/*W*_0_. The mechanical tests were performed using the Exceed E42 electronic universal testing machine from MTS Systems Corporation. At least three specimens were tested and averaged for each sample. The deflection rate was 50 mm min^−1^. Engineering stress (σ_E_) is the applied load on the sample divided by the initial cross-sectional area. Assuming constant volume, stress (σ) was calculated by multiplying σ_E_ by a factor of (1 + *λ*), where *λ* is the strain. Toughness is defined as an integral area under stress-strain curve.

## Supplementary information

Supplementary Information

Peer Review File

## Data Availability

The authors declare that data supporting the findings of this study are available within the paper and its Supplementary Information Files. Data generated in this study are provided in the Source Data file with this paper. Data are also available from the corresponding author upon request. Source data are provided with this paper.

## Source data

Source Data
