# Supplementary material for: Adaptable covalently cross-linked fibers
Source: Nat Commun. 2023 Apr 18;14:2218. doi: 10.1038/s41467-023-37850-w (PMC10113382; doi:10.1038/s41467-023-37850-w)
Supplement: Supplementary file 1 — Supplementary Information [file 41467_2023_37850_MOESM1_ESM.pdf]

Supplementary Information

Adaptable covalently cross-linked fibers

Hui Tan[1]†, Luzhi Zhang[1,2]†, Xiaopeng Ma[1], Lijie Sun[1,2], Dingle Yu[1], Zhengwei You[2]*

[1] Respiratory Department, Shenzhen Children's Hospital, Shenzhen, 518038, China

[2] State Key Laboratory for Modification of Chemical Fibers and Polymer Materials, College of Materials Science and Engineering, Institute of Functional Materials, Research Base of Textile Materials for Flexible Electronics and Biomedical Applications (China Textile Engineering Society), Shanghai Engineering Research Center of Nano-Biomaterials and Regenerative Medicine, Donghua University, Shanghai, 201620, China

† These authors contributed equally: Hui Tan, Luzhi Zhang.

* E-mail: zyou@dhu.edu.cn

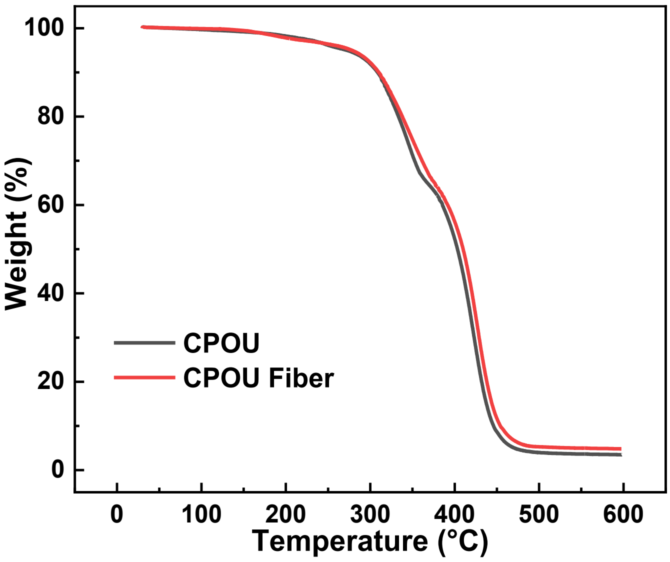

Supplementary Figure 1. TGA curves of synthesized CPOU and CPOU fiber.

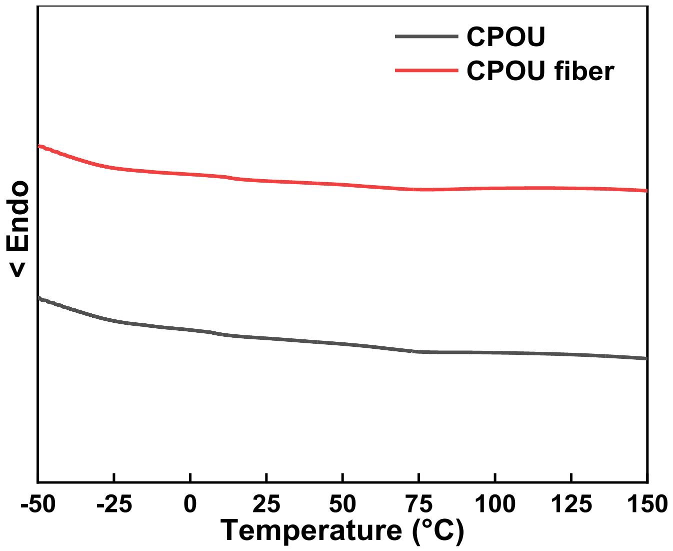

Supplementary Figure 2. DSC curves of synthesized CPOU and CPOU fiber.

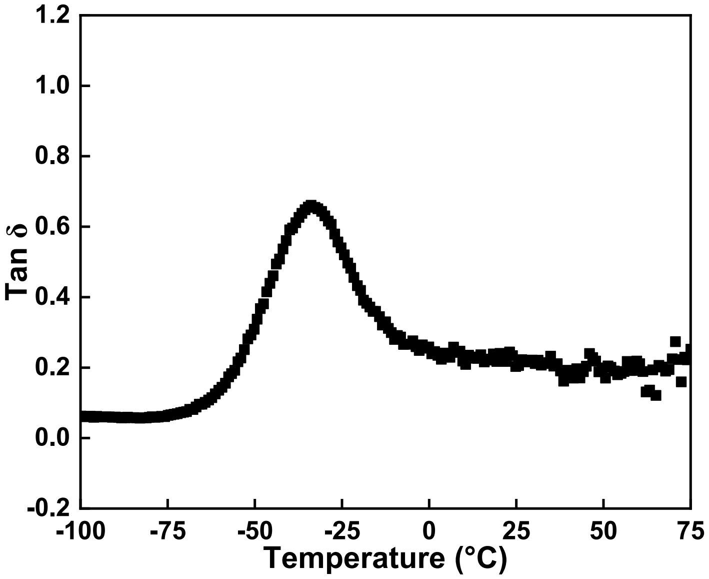

Supplementary Figure 3. Temperature-dependent curve of loss factor for CPOU.

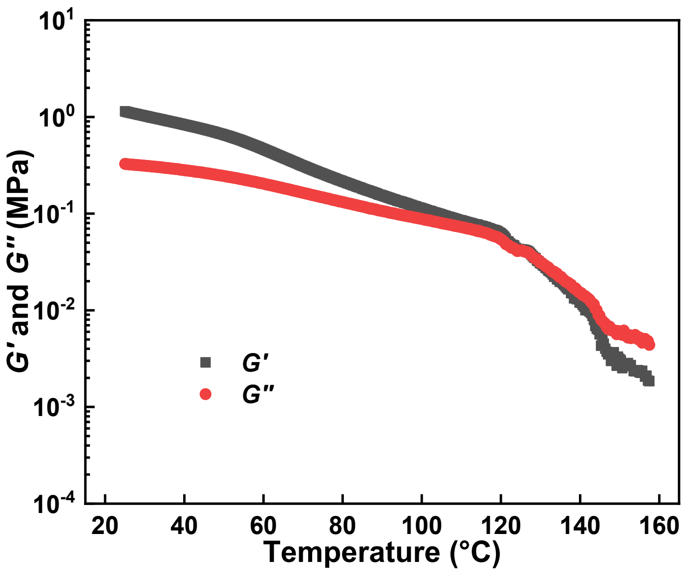

Supplementary Figure 4. Temperature-dependent curves of storage modulus and loss modulus for TPOU.

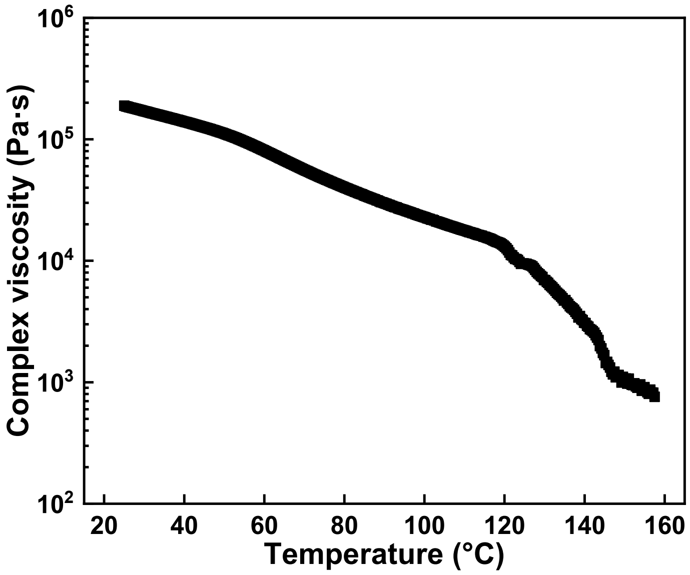

Supplementary Figure 5. Temperature-dependent curves of complex viscosity for TPOU.

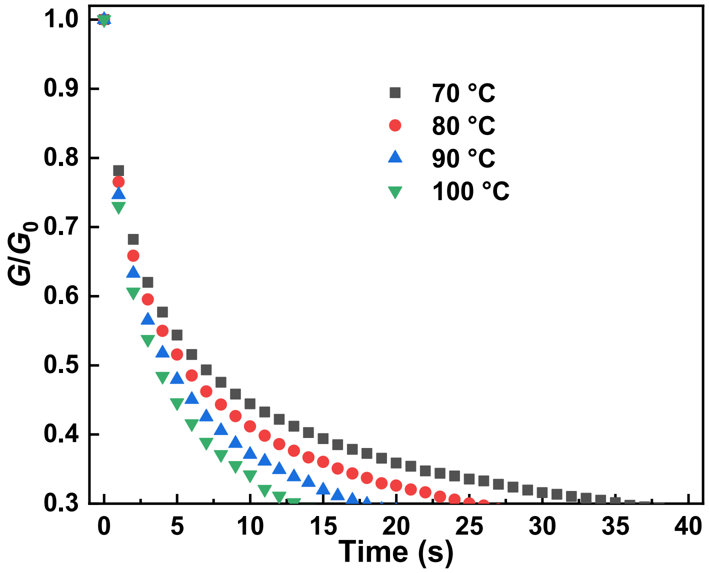

Supplementary Figure 6. Temperature-dependent stress-relaxation curves of TPOU.

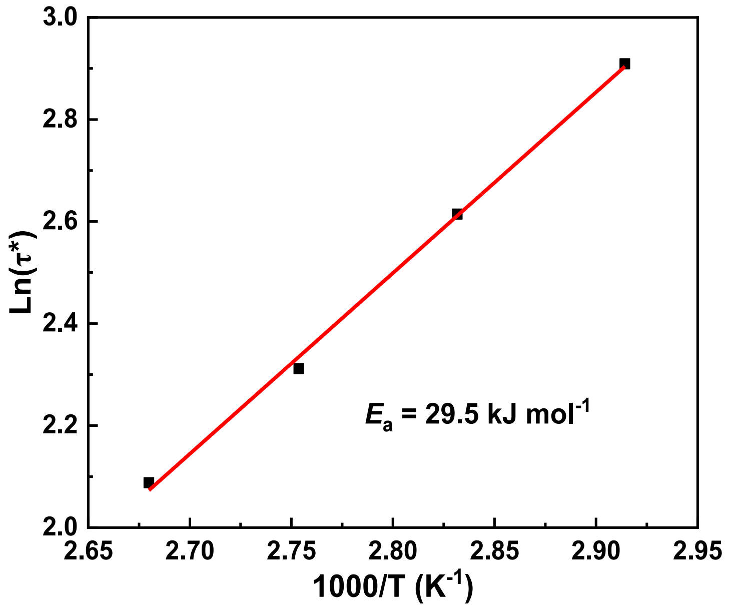

Supplementary Figure 7. Ln(τ*) plotted to 1000/T curves for TPOU and the linear fitted lines with the corresponding apparent activation energy.

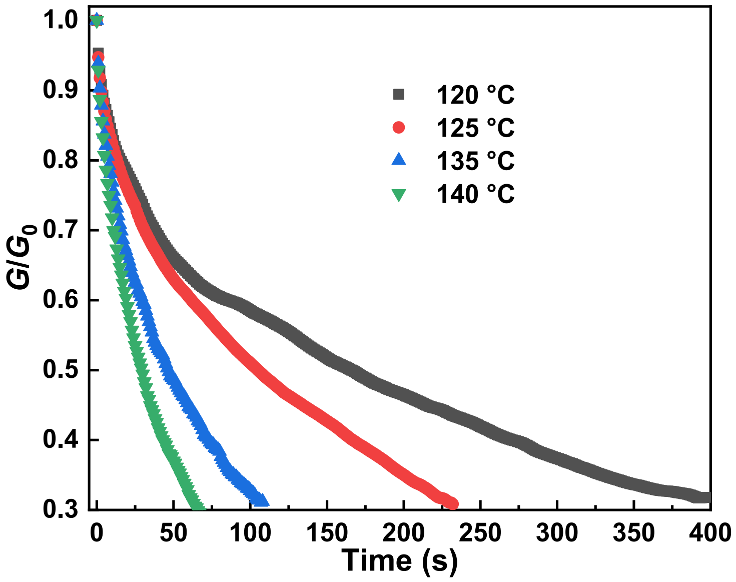

Supplementary Figure 8. Temperature-dependent stress-relaxation curves of CPOU.

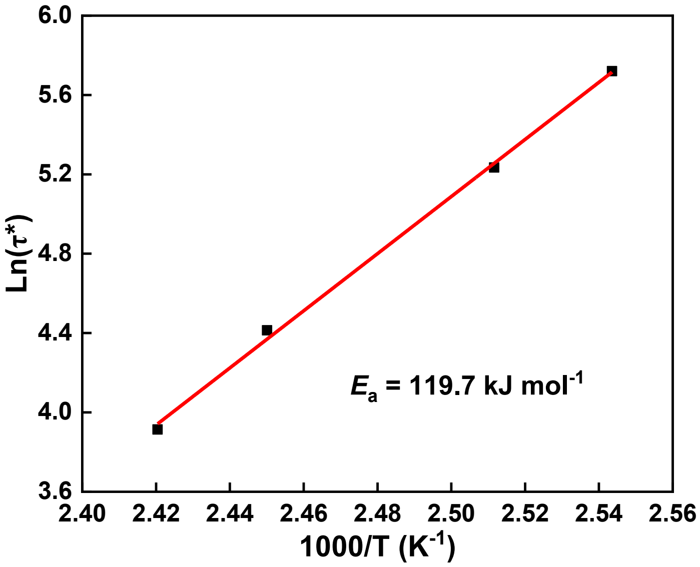

Supplementary Figure 9. Ln(τ*) plotted to 1000/T curves for CPOU and the linear fitted lines with the corresponding apparent activation energy.

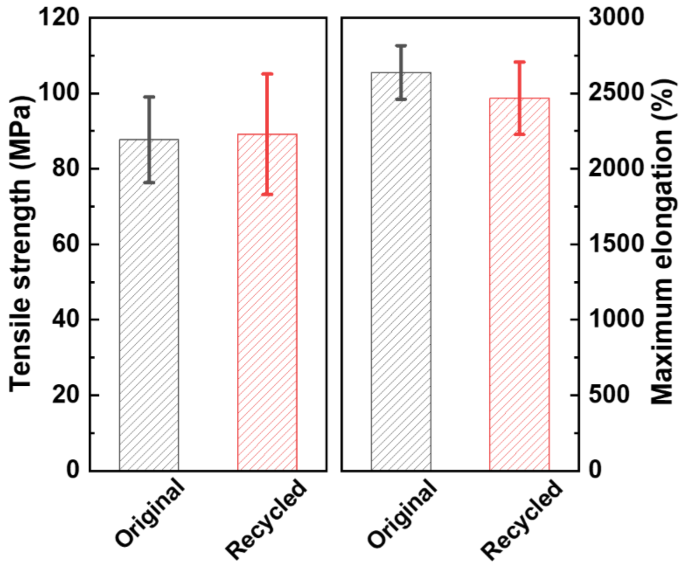

Supplementary Figure 10. Comparison of tensile strength and maximum elongation of original and recycled CPOU fibers (error bars: standard deviations).

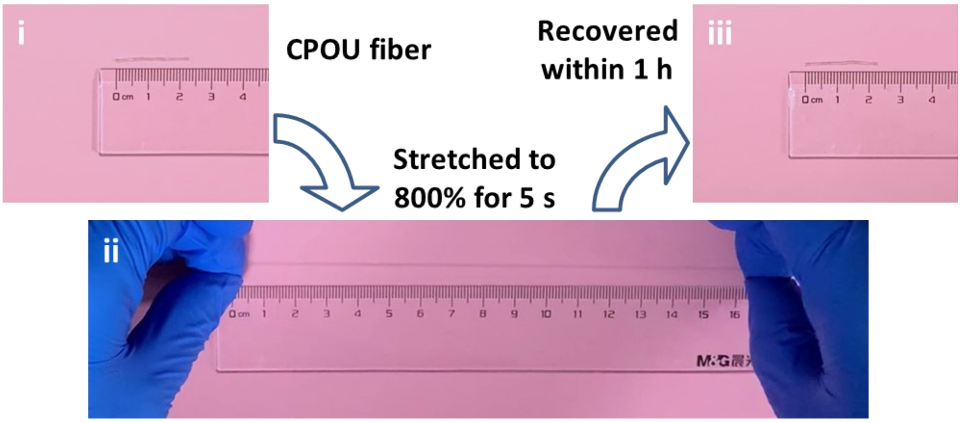

Supplementary Figure 11. Photographs of (i) original, (ii) 8 times stretched, and (iii)1 h recovered (at room temperature) CPOU fiber.

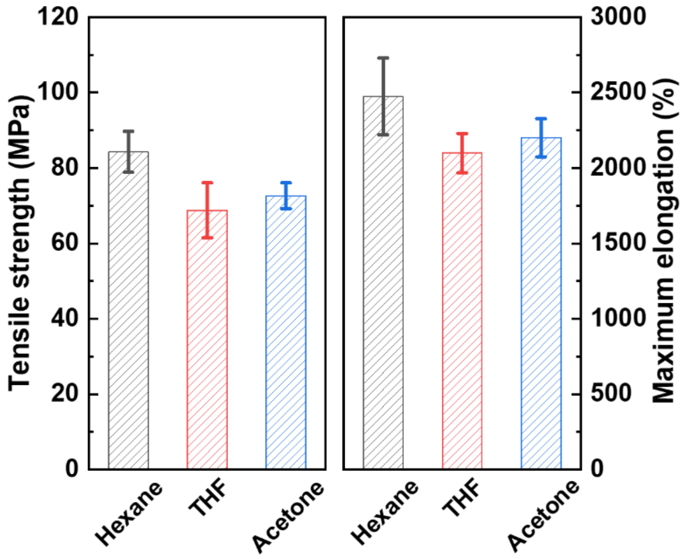

Supplementary Figure 12. Tensile strength and maximum elongation of dried CPOU fiber after swelling with different organic solvents (error bars: standard deviations).

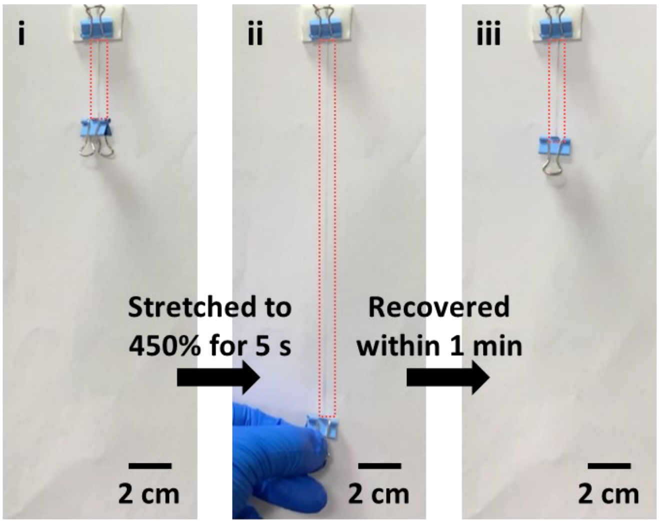

Supplementary Figure 13. Photographs of dried CPOU fiber after swelling with THF: (i) lifting an object, (ii) 4.5 times stretched, and (iii) recovered (residual deformation: 20%) within 1 min.

Supplementary Table 1. Comparison of different fibers.

	Fibers	Method	Tensile strength (MPa)	Maximum elongation (%)	Ref.
**Covalently cross-linked fibers**	CPOU	Melt-spinning	87.7	2639	This work
	Gel	Microfluidic spinning & photo-curing	0.9	500	1
	Gel	Wet-spinning & photo-curing	1	400	2
	Gel	Wet-spinning & photo-curing	0.66-1.08	35-65	3
	Liquid crystal polymer	Melt-spinning & curing in crosslinker solution	16.5-26.3	5-8	4
	Liquid crystal polymer	Mold-processing	96	1500	5
**Non-covalently cross-linked fibers**	Polyurethane	Wet-spinning	55	450	6
	Polyurethane	Wet-spinning	87.3	520	7
	Polyurethane	Wet-spinning	34.7	320	8
	Polyurethane	Wet-spinning	34.1	520	9
	Polyurethane/ polycaprolactone	Wet-spinning	6.2	310	10

Supplementary Table 2. Swelling ratio of CPOU fibers in various solvents.

Solvent*	Swelling ratio (%)
Hexane	101 ± 1
Tetrahydrofuran	235 ± 39
Acetone	230 ± 28
Ethanol	103 ± 2
Dimethylformamide	288 ± 30
Dimethyl sulfoxide	112 ± 4
Water	102 ± 2

*Ranked by the polarity.

Supplementary References

1. Kim, D., Ahn, S.-K. & Yoon, J. Highly Stretchable Strain Sensors Comprising Double Network Hydrogels Fabricated by Microfluidic Devices. *Adv. Mater. Technol.* **4**, 1800739 (2019).
2. Song, J. *et al.* Mechanically and Electronically Robust Transparent Organohydrogel Fibers. *Adv. Mater.* **32**, 1906994 (2020).
3. Chen, G. *et al.* Integrated dynamic wet spinning of core-sheath hydrogel fibers for optical-to-brain/tissue communications. *Natl. Sci. Rev.* **8**, nwaa209 (2021).
4. Guo, C., Gao, J., Ma, S. & Zhang, H. Efficient preparation of chemically crosslinked recyclable photodeformable azobenzene polymer fibers with high processability and reconstruction ability via a facile post-crosslinking method. *Eur. Polym. J.* **139**, 109998 (2020).
5. Yao, M., Wu, B., Feng, X., Sun, S. & Wu, P. A Highly Robust Ionotronic Fiber with Unprecedented Mechanomodulation of Ionic Conduction. *Adv. Mater.* **33**, 2103755 (2021).
6. He, Z. *et al.* Effect of MWCNT content on the mechanical and strain-sensing performance of Thermoplastic Polyurethane composite fibers. *Carbon* **146**, 701-708 (2019).
7. Wu, G.-Q. *et al.* Highly Stretchable and Conductive Hybrid Fibers for High-performance Fibrous Electrodes and All-solid-state Supercapacitors. *Chinese J. Polym. Sci.* **38**, 531-539 (2020).
8. Liu, J. *et al.* A highly stretchable and ultra-sensitive strain sensing fiber based on a porous core–network sheath configuration for wearable human motion detection. *Nanoscale* **14**, 12418-12430 (2022).
9. Li, W., Xu, L., Wang, X., Zhu, R. & Yan, Y. Phase Change Energy Storage Elastic Fiber: A Simple Route to Personal Thermal Management. *Polymers* **14**, 53 (2022).
10. Qu, M. *et al.* A thermally-electrically double-responsive polycaprolactone – thermoplastic polyurethane/multi-walled carbon nanotube fiber assisted with highly effective shape memory and strain sensing performance. *Chem. Eng. J.* **427**, 131648 (2022).
