# Supplementary material for: Adaptable covalently cross-linked fibers
Source: Nat Commun. 2023 Apr 18;14:2218. doi: 10.1038/s41467-023-37850-w (PMC10113382; doi:10.1038/s41467-023-37850-w)
Supplement: Supplementary file 2 — Peer Review File [file 41467_2023_37850_MOESM2_ESM.pdf]

nature portfolio

Peer Review File

Adaptable covalently cross-linked fibers

Open Access This file is licensed under a Creative Commons Attribution 4.0 International License, which permits use, sharing, adaptation, distribution and reproduction in any medium or format, as long as you give appropriate credit to the original author(s) and the source, provide a link to the Creative Commons license, and indicate if changes were made. In the cases where the authors are anonymous, such as is the case for the reports of anonymous peer reviewers, author attribution should be to 'Anonymous Referee' followed by a clear attribution to the source work. The images or other third party material in this file are included in the article's Creative Commons license, unless indicated otherwise in a credit line to the material. If material is not included in the article's Creative Commons license and your intended use is not permitted by statutory regulation or exceeds the permitted use, you will need to obtain permission directly from the copyright holder. To view a copy of this license, visit http://creativecommons.org/licenses/by/4.0/.

Reviewers' Comments:

Reviewer #1:
Remarks to the Author:
The manuscript presents results on the preparation and fiber spinning of covalent adaptable networks (CANs) based on dynamic oxime-urethane crosslinks. This concept opens up for chemical recycling of crosslinked fibers, which are otherwise typically difficult to reuse. However, the concept of CANs is well-known and quite extensively studied, and the dynamic features of oxime-urethane crosslinks have also been previously reported on.

The major achievement and novelty in the present work is the use of CANs to melt spin fibers to open up for recycling of crosslinked fiber materials. Deformation by creep under load is to some degree inherent due to the bond dynamics of CANs and is a well-known limitation for this class of materials in many applications. Conventional covalent crosslinking of fibers is often carried out in the industry to prevent creep, which causes sag of the textile. As a consequence, any recycling of these textiles is very challenging. In the present study the authors benchmark their CAN fibers against a corresponding non-crosslinked fiber. It would have been much more interesting to compare with a corresponding conventionally (non-dynamically) crosslinked fiber to see how much the properties are influenced by the dynamic nature of the oxime-urethane crosslinks. After proper attention to the comments, this paper may be interesting for researchers in the textile and fiber area.

1. The authors must provide full details and descriptions of their measurements and results. Throughout the paper the authors describe experimental results without giving full details, e.g., on line 164-166. Here it is state that the fiber was "stretched to 8 times its original length and almost recovered to its original dimensions after relaxing for 1 h at room temperature" (Figure 3a). This result must be highly dependent on how long time the fiber was kept in the stretched position, which is not given. Moreover, on line 185-186: "The dried CPOU fiber after swelling in THF was highly stretchable and could immediately recover after being stretched to about 4.5 times its original length". However, in Figure 4b, there seems to be a noticeable residual deformation after the stretching. For how long was the fiber stretched, and how large was the residual deformation in this case?
2. How repeatable are the melt spinning results and the measured data reported in the paper? There are hardly any error bars
3. It would strengthen the significance of their work if the authors could demonstrate by re-spinning of chemically recycled CANs.
4. Minor point: the authors may consider to change the title to "Cross-linked fibers from covalent adaptable networks".

Reviewer #2:
Remarks to the Author:
This manuscript describes the synthetic investigations of a poly(oxime-urethane) fibre for stretchable electronics. The reversible nature of the chemical bonds in the polymers enable melt-spinning process, which can be more sustainable compared to wet-spinning process (currently widely used to prepare elastic fibres). The obtained fibre exhibits good solvent resistance and excellent tensile properties. I believe that this manuscript has sufficient novelty and high potential to improve the state-of-the-art fabrication of elastic fibres like Lycra, Spandex etc. Nevertheless, I have some questions before I can recommend publication:
1. I suggest to compare the mechanical properties of the obtained fibre with the data for other commercial fibres (e.g. elastane, elastollan etc) or literature reports to clarify the discussions.
2. Regarding solvent resistance studies and Table S1, I suggest providing the swelling ratio of the fibres. It should be more informative than just giving +/- signs as shown in Table S1. Also other organic solvents with ideally tuneable polarity should be investigated in addition to THF.
3. In the manuscript, it was written that the fibre recovers completely from stretching to 800 % after

1 hour. Then it recovers “immediately” after being stretched 4.5 times after being submerged in THF and then dried. Finally, it recovers instantly after being stretched 2.5 times while being submerged in THF. It is difficult to understand by using different stretching lengths in these cases. I suggest stretching the fibre to the same length each time for better comparison. Also elongation at break maybe interesting to measure.

Response to Reviewers

Note:

- **The revised text in manuscript has been highlighted in yellow.**
- **In this letter, the reviewer comments and answers (highlighted by blue) are listed one by one.**

Reviewer #1:

The manuscript presents results on the preparation and fiber spinning of covalent adaptable networks (CANs) based on dynamic oxime-urethane crosslinks. This concept opens up for chemical recycling of crosslinked fibers, which are otherwise typically difficult to reuse. However, the concept of CANs is well-known and quite extensively studied, and the dynamic features of oxime-urethane crosslinks have also been previously reported on.

The major achievement and novelty in the present work is the use of CANs to melt spin fibers to open up for recycling of crosslinked fiber materials. Deformation by creep under load is to some degree inherent due to the bond dynamics of CANs and is a well-known limitation for this class of materials in many applications. Conventional covalent crosslinking of fibers is often carried out in the industry to prevent creep, which causes sag of the textile. As a consequence, any recycling of these textiles is very challenging. In the present study the authors benchmark their CAN fibers against a corresponding non-crosslinked fiber. It would have been much more interesting to compare with a corresponding conventionally (non-dynamically) crosslinked fiber to see how much the properties are influenced by the dynamic nature of the oxime-urethane crosslinks. After proper attention to the comments, this paper may be interesting for researchers in the textile and fiber area.

Answer: We greatly appreciate the positive and constructive comments. According to the suggestion, we have conducted further recycling experiments of CPOU fiber and detailed them in comment 3. Compared with most traditional fibers, one of the outstanding features of CPOU fiber is its convenient processability. Conventionally (non-dynamically) crosslinked polymers are difficult to process in the same way as CPOU and may damage processing equipment. We have compared CPOU fibers with reported covalently crosslinked fibers without dynamic covalent bonds, and added the results in the main text: "The polymeric networks, preparation methods, and mechanical properties of reported fibers were summarized in Supplementary Table 1. For covalently crosslinked fibers: some gel fibers could be continuously prepared by microfluidic spinning/wet-spinning and post-crosslinking, but their mechanical properties (tensile strength ~ 1 MPa) might be difficult to meet the needs of many practical use scenarios; liquid crystal polymer fibers were prepared by wet-spinning/mold-processing and post-crosslinking, so it was difficult to achieve continuous preparation. For non-covalently crosslinked fibers (especially polyurethane fibers), they usually showed appreciable tensile strength (34-87 MPa) but limited maximum elongation (320%-520%). Most of polyurethane fibers were prepared by wet-spinning, which required

the use of organic solvents (e.g., dimethylformamide) and additional post-treatment processes (e.g., solvent removal). In this work, the spinning process of covalently crosslinked CPOU fibers did not require solvents and achieved continuous preparation. In addition, the tensile strength (87.7 MPa) of CPOU fibers exceeded most of the reported fibers, and showed superior maximum elongation (2639%).”

Supplementary Table 1. Comparison of different fibers.

	**Fibers**	**Method**	**Tensile strength (MPa)**	**Maximum elongation (%)**	**Ref.**
**Covalently cross-linked fibers**	CPOU	Melt-spinning	87.7	2639	This work
	Gel	Microfluidic spinning & photo-curing	0.9	500	1
	Gel	Wet-spinning & photo-curing	1	400	2
	Gel	Wet-spinning & photo-curing	0.66-1.08	35-65	3
	liquid crystal polymer	Melt-spinning & curing in crosslinker solution	16.5-26.3	5-8	4
	liquid crystal polymer	Mold-processing	96	1500	5
**Non-covalently cross-linked fibers**	Polyurethane	Wet-spinning	55	450	6
	Polyurethane	Wet-spinning	87.3	520	7
	Polyurethane	Wet-spinning	34.7	320	8
	Polyurethane	Wet-spinning	34.1	520	9
	Polyurethane/ polycaprolactone	Wet-spinning	6.2	310	10

References

1. Kim, D., Ahn, S.-K. & Yoon, J. Highly Stretchable Strain Sensors Comprising Double Network Hydrogels Fabricated by Microfluidic Devices. *Adv. Mater. Technol.* **4**, 1800739 (2019).

2. Song, J. *et al.* Mechanically and Electronically Robust Transparent Organohydrogel Fibers. *Adv. Mater.* **32**, 1906994 (2020).

3. Chen, G. *et al.* Integrated dynamic wet spinning of core-sheath hydrogel fibers for optical-to-brain/tissue communications. *Natl. Sci. Rev.* **8**, nwaa209 (2021).

4. Guo, C., Gao, J., Ma, S. & Zhang, H. Efficient preparation of chemically crosslinked recyclable photodeformable azobenzene polymer fibers with high processability and reconstruction ability via a facile post-crosslinking method. *Eur. Polym. J.* **139**, 109998 (2020).

5. Yao, M., Wu, B., Feng, X., Sun, S. & Wu, P. A Highly Robust Ionotronic Fiber with Unprecedented Mechanomodulation of Ionic Conduction. *Adv. Mater.* **33**, 2103755 (2021).

6. He, Z. *et al.* Effect of MWCNT content on the mechanical and strain-sensing performance of Thermoplastic Polyurethane composite fibers. *Carbon* **146**, 701-708 (2019).
7. Wu, G.-Q. *et al.* Highly Stretchable and Conductive Hybrid Fibers for High-performance Fibrous Electrodes and All-solid-state Supercapacitors. *Chinese J. Polym. Sci.* **38**, 531-539 (2020).
8. Liu, J. *et al.* A highly stretchable and ultra-sensitive strain sensing fiber based on a porous core–network sheath configuration for wearable human motion detection. *Nanoscale* **14**, 12418-12430 (2022).
9. Li, W., Xu, L., Wang, X., Zhu, R. & Yan, Y. Phase Change Energy Storage Elastic Fiber: A Simple Route to Personal Thermal Management. *Polymers* **14**, 53 (2022).
10. Qu, M. *et al.* A thermally-electrically double-responsive polycaprolactone – thermoplastic polyurethane/multi-walled carbon nanotube fiber assisted with highly effective shape memory and strain sensing performance. *Chem. Eng. J.* **427**, 131648 (2022).

1. The authors must provide full details and descriptions of their measurements and results. Throughout the paper the authors describe experimental results without giving full details, e.g., on line 164-166. Here it is state that the fiber was “stretched to 8 times its original length and almost recovered to its original dimensions after relaxing for 1 h at room temperature” (Fig. 3a). This result must be highly dependent on how long time the fiber was kept in the stretched position, which is not given. Moreover, on line 185-186: “The dried CPOU fiber after swelling in THF was highly stretchable and could immediately recover after being stretched to about 4.5 times its original length”. However, in Fig. 4b, there seems to be a noticeable residual deformation after the stretching. For how long was the fiber stretched, and how large was the residual deformation in this case?

Answer: Many thanks for the kind and professional suggestion. We have added all the details in the main text and revised relevant figures: “the CPOU fiber was stretched to 8 times its original length for 5 s and almost recovered to its original dimension after relaxing for 1 h at room temperature (Supplementary Fig. 11)”, “the dried CPOU fiber after swelling with THF was recovered (residual deformation: 20%) within 1 min after being stretched to 4.5 times its original length for 5 s (Supplementary Fig. 13).”

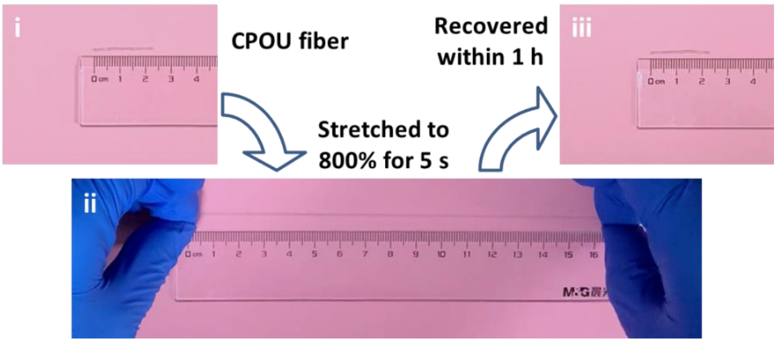

Supplementary Figure 11. Photographs of (i) original, (ii) 8 times stretched, and (iii) 1 h recovered (at room temperature) CPOU fiber.

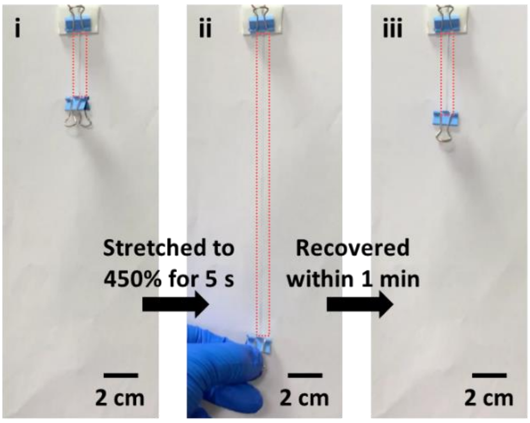

Supplementary Figure 13. Photographs of dried CPOU fiber after swelling with THF: (i) lifting an object, (ii) 4.5 times stretched, and (iii) recovered (residual deformation: 20%) within 1 min.

2. How repeatable are the melt spinning results and the measured data reported in the paper? There are hardly any error bars

Answer: Many thanks for the important point. We have performed multiple melt spinning, and at least three specimens were tested (e.g., tensile test) and averaged for each sample, demonstrating the reproducibility of the results. We have added related data with error bars in the main text and figures: "The mechanical tests were performed using the Exceed E42 electronic universal testing machine from MTS Systems Corporation. At least three specimens were tested and averaged for each sample.", "Fig. 3b, the tensile strength of CPOU fiber (87.68 ± 11.34 MPa) was more than 28 times that of TPOU fiber (3.13 ± 0.05 MPa), while the maximum elongation of CPOU fiber (2639% ± 178%) was 3.3 times that of TPOU fiber (795% ± 40%). The toughness of CPOU fiber (1187 ± 227 MJ m^{-3}) was more than 74 times that of TPOU fiber (16 ± 1 MJ m^{-3})."

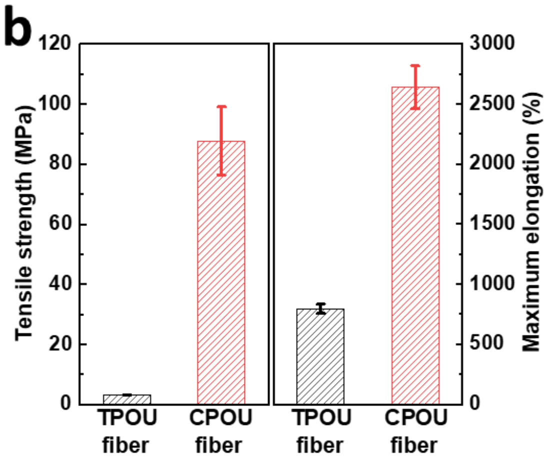

Figure 3. Mechanical properties of TPOU and CPOU fibers. (b) Comparison of their tensile strength and maximum elongation.

"Compared with original CPOU fibers, the mechanical properties of the recycled CPOU fibers were basically maintained (Supplementary Fig. 10)"

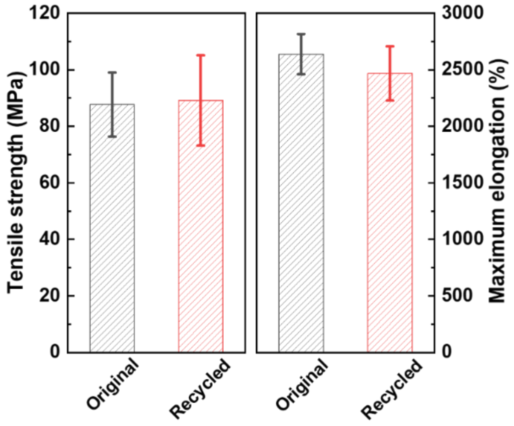

Supplementary Figure 10. Comparison of tensile strength and maximum elongation of original and recycled CPOU fibers.

3. It would strengthen the significance of their work if the authors could demonstrate by re-spinning of chemically recycled CANs.

Answer: Many thanks for the professional suggestion. According to the suggestion, we have now conducted further experiments, and added the results in main text: "Owing to the dynamic nature of the CPOU networks, CPOU fibers can be recycled and melt-spun again. Compared with original CPOU fibers, the mechanical properties of the recycled CPOU fibers were basically maintained (Supplementary Fig. 10), demonstrating the recyclability of CPOU fibers."

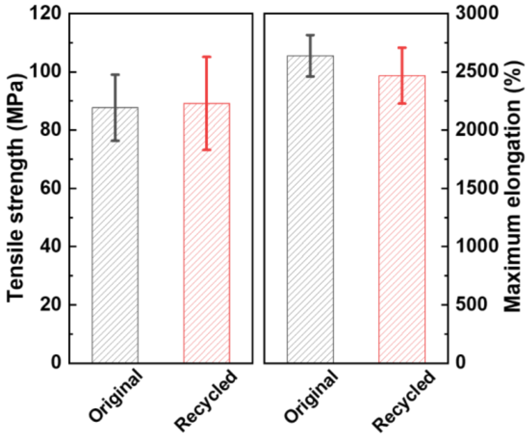

Supplementary Figure 10. Comparison of tensile strength and maximum elongation of original and recycled CPOU fibers.

4. Minor point: the authors may consider to change the title to "Cross-linked fibers from covalent adaptable networks".

Answer: Many thanks for the good point. We have revised the title accordingly.

Reviewer #2:

This manuscript describes the synthetic investigations of a poly(oxime-urethane) fibre for stretchable electronics. The reversible nature of the chemical bonds in the polymers enable melt-spinning process, which can be more sustainable compared to wet-spinning process (currently widely used to prepare elastic fibres). The obtained fibre exhibits good solvent resistance and excellent tensile properties. I believe that this manuscript has sufficient novelty and high potential to improve the state-of-the-art fabrication of elastic fibres like Lycra, Spandex etc. Nevertheless, I have some questions before I can recommend publication:

Answer: We greatly appreciate the positive and constructive comments. We have addressed all your questions one by one as below.

1. I suggest to compare the mechanical properties of the obtained fibre with the data for other commercial fibres (e.g. elastane, elastollan etc) or literature reports to clarify the discussions.

Answer: Many thanks for the professional suggestion. We have compared CPOU fibers with other fibers (including elastollan in Ref. 6-9), and added the results in the main text: "The polymeric networks, preparation methods, and mechanical properties of reported fibers were summarized in Supplementary Table 1. For covalently crosslinked fibers: some gel fibers could be continuously prepared by microfluidic spinning/wet-spinning and post-crosslinking, but their mechanical properties (tensile strength ~ 1 MPa) might be difficult to meet the needs of many practical use scenarios; liquid crystal polymer fibers were prepared by wet-spinning/mold-processing and post-crosslinking was, so it was difficult to achieve continuous preparation. For non-covalently crosslinked fibers (especially polyurethane fibers), they usually showed appreciable tensile strength (34-87 MPa) but limited maximum elongation (320%-520%). Most of polyurethane fibers were prepared by wet-spinning, which required the use of organic solvents (e.g., dimethylformamide) and additional post-treatment processes (e.g., solvent removal). In this work, the spinning process of covalently crosslinked CPOU fibers did not require solvents and achieved continuous preparation. In addition, the tensile strength (87.7 MPa) of CPOU fibers exceeded most of the reported fibers, and showed superior maximum elongation (2639%)."

Supplementary Table 1. Comparison of different fibers.

	**Fibers**	**Method**	**Tensile strength (MPa)**	**Maximum elongation (%)**	**Ref.**
**Covalently cross-linked fibers**	CPOU	Melt-spinning	87.7	2639	This work
	Gel	Microfluidic spinning & photo-curing	0.9	500	1
	Gel	Wet-spinning & photo-curing	1	400	2

	Gel	Wet-spinning & photo-curing	0.66-1.08	35-65	3
	liquid crystal polymer	Melt-spinning & curing in crosslinker solution	16.5-26.3	5-8	4
	liquid crystal polymer	Mold-processing	96	1500	5
**Non-covalently cross-linked fibers**	Polyurethane	Wet-spinning	55	450	6
	Polyurethane	Wet-spinning	87.3	520	7
	Polyurethane	Wet-spinning	34.7	320	8
	Polyurethane	Wet-spinning	34.1	520	9
	Polyurethane/ polycaprolactone	Wet-spinning	6.2	310	10

References

1. Kim, D., Ahn, S.-K. & Yoon, J. Highly Stretchable Strain Sensors Comprising Double Network Hydrogels Fabricated by Microfluidic Devices. *Adv. Mater. Technol.* **4**, 1800739 (2019).

2. Song, J. *et al*. Mechanically and Electronically Robust Transparent Organohydrogel Fibers. *Adv. Mater.* **32**, 1906994 (2020).

3. Chen, G. *et al*. Integrated dynamic wet spinning of core-sheath hydrogel fibers for optical-to-brain/tissue communications. *Natl. Sci. Rev.* **8**, nwaa209 (2021).

4. Guo, C., Gao, J., Ma, S. & Zhang, H. Efficient preparation of chemically crosslinked recyclable photodeformable azobenzene polymer fibers with high processability and reconstruction ability via a facile post-crosslinking method. *Eur. Polym. J.* **139**, 109998 (2020).

5. Yao, M., Wu, B., Feng, X., Sun, S. & Wu, P. A Highly Robust Ionotronic Fiber with Unprecedented Mechanomodulation of Ionic Conduction. *Adv. Mater.* **33**, 2103755 (2021).

6. He, Z. *et al*. Effect of MWCNT content on the mechanical and strain-sensing performance of Thermoplastic Polyurethane composite fibers. *Carbon* **146**, 701-708 (2019).

7. Wu, G.-Q. *et al*. Highly Stretchable and Conductive Hybrid Fibers for High-performance Fibrous Electrodes and All-solid-state Supercapacitors. *Chinese J. Polym. Sci.* **38**, 531-539 (2020).

8. Liu, J. *et al*. A highly stretchable and ultra-sensitive strain sensing fiber based on a porous core–network sheath configuration for wearable human motion detection. *Nanoscale* **14**, 12418-12430 (2022).

9. Li, W., Xu, L., Wang, X., Zhu, R. & Yan, Y. Phase Change Energy Storage Elastic Fiber: A Simple Route to Personal Thermal Management. *Polymers* **14**, 53 (2022).

10. Qu, M. *et al*. A thermally-electrically double-responsive polycaprolactone – thermoplastic polyurethane/multi-walled carbon nanotube fiber assisted with highly effective shape memory and strain sensing performance. *Chem. Eng. J.* **427**, 131648 (2022).

2. Regarding solvent resistance studies and Table S1, I suggest providing the swelling ratio of the fibres. It should be more informative than just giving +/- signs as shown in Table S1. Also

other organic solvents with ideally tuneable polarity should be investigated in addition to THF.

Answer: Many thanks for the professional suggestion. According to the suggestion, we have conducted further experiments, and added hexane with less polarity than THF as a new solvent. We have added the results in main text: "According to dissolution experiments, CPOU fibers were insoluble in common solvents, and the swelling ratios were summarized in Supplementary Table 2."

Supplementary Table 2. Swelling ratio of CPOU fibers in various solvents.

Solvent*	Swelling ratio (%)
Hexane	101 ± 1
Tetrahydrofuran	235 ± 39
Acetone	230 ± 28
Ethanol	103 ± 2
Dimethylformamide	288 ± 30
Dimethyl sulfoxide	112 ± 4
Water	102 ± 2

*Ranked by the polarity.

3. In the manuscript, it was written that the fibre recovers completely from stretching to 800 % after 1 hour. Then it recovers "immediately" after being stretched 4.5 times after being submerged in THF and then dried. Finally, it recovers instantly after being stretched 2.5 times while being submerged in THF. It is difficult to understand by using different stretching lengths in these cases. I suggest stretching the fibre to the same length each time for better comparison. Also elongation at break maybe interesting to measure.

Answer: Many thanks for the kind and professional suggestion. According to the suggestion, we have conducted further experiments to keep the stretching ratio consistent (2.5 times) and measure the maximum elongation of dried CPOU fiber after swelling with solvents. We have added the results in main text: "As a demonstration, CPOU fiber was stretched to 2.5 times its original length for 5 s and almost recovered to its original dimension after relaxing for 1 min at room temperature (Fig. 3e)."

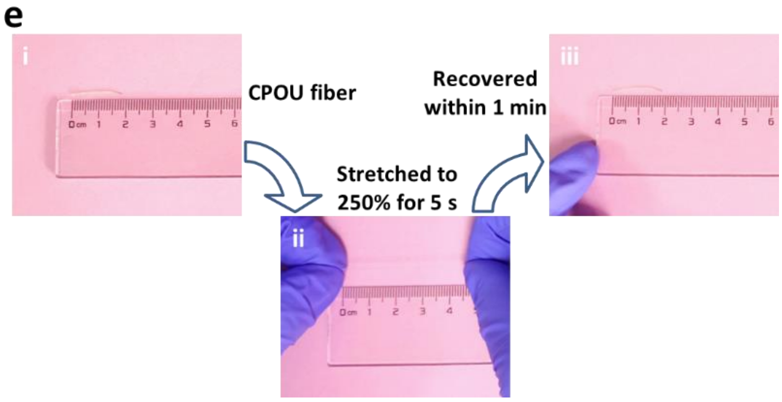

Figure 3. (e) Photographs of (i) original, (ii) 2.5 times stretched, and (iii)1 min recovered (at room temperature) CPOU fiber.

"The dried CPOU fiber after swelling with THF could almost recover within 1 min after being stretched to 2.5 times its original length for 5 s (Fig. 4b)"

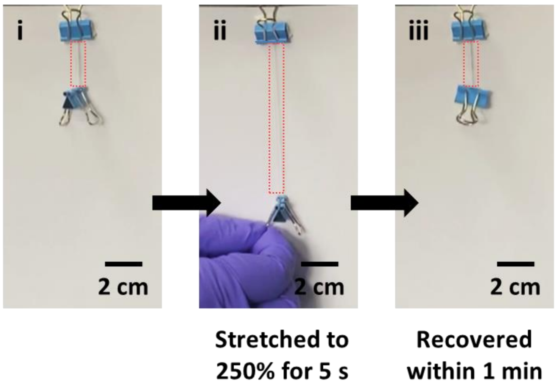

Figure 4. (b) Photographs of dried CPOU fiber after swelling with THF: (i) lifting an object, (ii) 2.5 times stretched, and (iii) recovered within 1 min.

"After being immersed in THF for 5 min, CPOU/copper fiber maintained high conductivity, stretchability and elasticity with an immediate (within 1 min) recovery to 1.06 times its original length after being 2.5 times stretched for 5 s (Fig. 5b)"

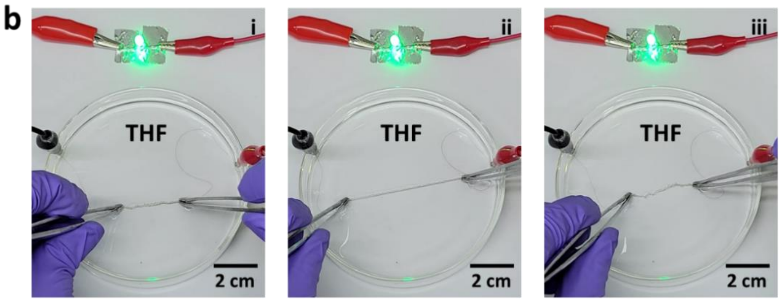

Figure 5. (b) Demonstration of the organic solvent resistant and stretchable properties for the CPOU/copper conductive fiber in series with an LED. (i) CPOU/copper conductive fiber immersed in THF, (ii) 2.5 times stretched for 5 s, and (iii) recovered within 1 min.

"Moreover, after the CPOU fibers were immersed in organic solvents with different polarities for 5 min and dried, the mechanical properties of the CPOU fibers were basically maintained (Supplementary Fig. 12), demonstrating the solvent resistance of the CPOU fibers."

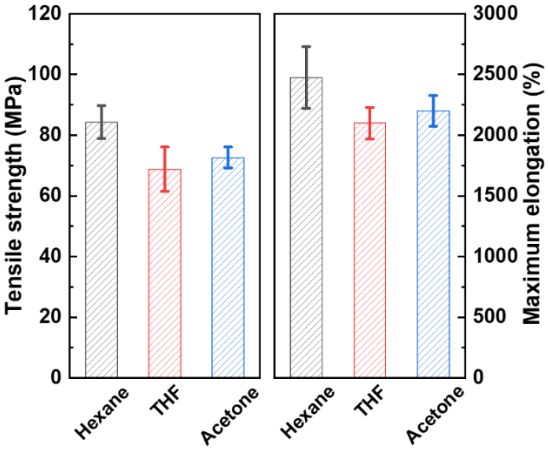

Supplementary Figure 12. Tensile strength and maximum elongation of dried CPOU fiber after swelling with different organic solvents.

Reviewers' Comments:

Reviewer #1:
Remarks to the Author:
The authors have responded well to all my comments and made the necessary additions and changes in the manuscript. My only remark on the revised version is that it is very difficult to see the fibers in Fig. 3e. Fibers are off course very thin, but can the authors improve the quality of the images?

Reviewer #2:
Remarks to the Author:
The authors have addressed all my concerns on the previous draft. Publication is recommended.

Response to Reviewers

Note:

- **The revised text in manuscript has been highlighted in yellow.**
- **In this letter, the reviewer comments and answers (highlighted by blue) are listed one by one.**

Reviewer #1:

The authors have responded well to all my comments and made the necessary additions and changes in the manuscript. My only remark on the revised version is that it is very difficult to see the fibers in Fig. 3e. Fibers are off course very thin, but can the authors improve the quality of the images?

Answer: We greatly appreciate the positive and constructive comments. According to the suggestion, we have improved the quality of Fig. 3e.

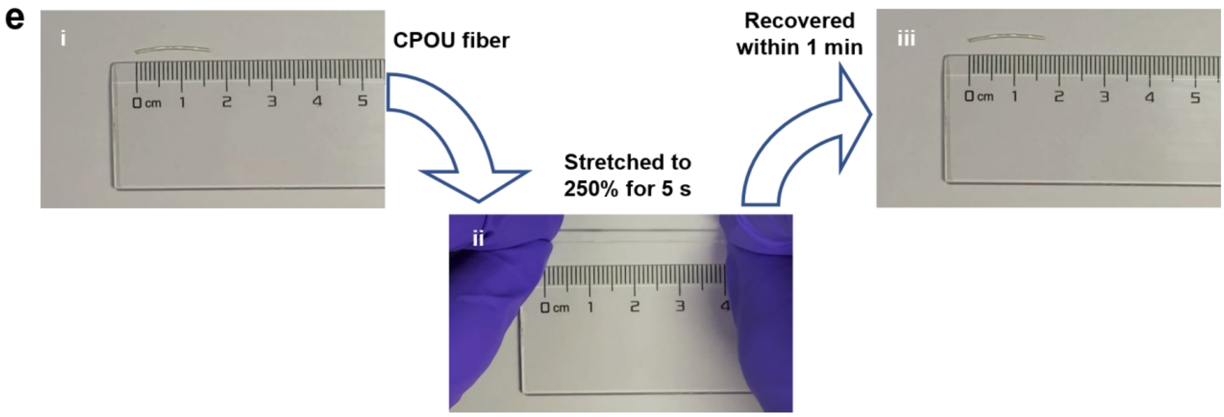

Fig. 3 (e) Photographs of (i) original, (ii) 2.5 times stretched, and (iii) 1 min recovered (at room temperature) CPOU fiber.

Reviewer #2:

The authors have addressed all my concerns on the previous draft. Publication is recommended.

Answer: Thank you very much for your previous great comments and recommendation for the publication of our manuscript.
